# The role of M1/M2 macrophage polarization in primary Sjogren’s syndrome

**DOI:** 10.1186/s13075-024-03340-7

**Published:** 2024-05-14

**Authors:** Xiaochan Chen, Linjiang Zhu, Huaxiang Wu

**Affiliations:** Department of Rheumatology, The Second Affiliated Hospital of Zhejiang University School of Medicine, No.88 Jiefang Road, Hangzhou, 310009 P.R. China

**Keywords:** Primary Sjogren’s syndrome, Macrophage polarization, Minor salivary gland

## Abstract

**Background:**

The purpose of this study was to investigate the role of macrophage polarization in the pathogenesis of primary Sjogren’s syndrome (pSS).

**Methods:**

Peripheral venous blood samples were collected from 30 patients with pSS and 30 healthy controls. Minor salivary gland samples were abtainted from 10 of these patients and 10 non-pSS controls whose minor salivary gland didn’t fulfill the classification criteria for pSS. Enzyme-linked immuno sorbent assay was used to examine the serum concentration of M1/M2 macrophage related cytokines (TNF-a, IL-6, IL-23, IL-4, IL-10 and TGF-β). Flow cytometry was used to examine the numbers of CD86^+^ M1 macrophages and CD206^+^ M2 macrophages in peripheral blood mononuclear cells (PBMCs). Immunofluorescence was used to test the infiltration of macrophages in minor salivary glands.

**Results:**

This study observed a significant increase in pSS patients both in the numbers of M1 macrophages in peripheral blood and serum levels of M1-related pro-inflammatory cytokines (IL-6, IL-23 and TNF-α). Conversely, M2 macrophages were downregulated in the peripheral blood of pSS patients. Similarly, in the minor salivary glands of pSS patients, the expression of M1 macrophages was increased, and that of M2 macrophages was decreased. Furthermore, a significantly positive correlation was found between the proportions of M1 macrophages in PBMCs and serum levels of IgG and RF.

**Conclusions:**

This study reveals the presence of an significant imbalance in M1/M2 macrophages in pSS patients. The M1 polarization of macrophages may play an central role in the pathogenesis of pSS.

**Supplementary Information:**

The online version contains supplementary material available at 10.1186/s13075-024-03340-7.

## Background

Primary Sjogren’s syndrome (pSS) is a chronic autoimmune disease characterized by extensive activation of immune cells and infiltration of lymphocytes in exocrine glands or other target organs. Salivary and lacrimal glands are the primarily affected exocrine glands. The principal clinical symptoms of pSS encompass xerostomia, xerophthalmia, fatigue, and arthralgia. A subset of patients may additionally exhibit extra-glandular multisystemic involvement. The impact of pSS on patients’ quality of life is substantial, despite the incomplete understanding of the underlying mechanisms of the disease.

Macrophages are a crucial component of the innate immune system and have significant involvement in processes such as inflammation, immune regulation, and tissue repair. While autoreactive T cells are recognized as primary contributors to the pathogenesis of pSS [1], it is also important to acknowledge the role of monocytes/macrophages in the development of this condition. Human studies have provided evidence of macrophage infiltration in the salivary glands of pSS patients, which has been correlated with disease severity [2, 3]. Analysis of single-cell RNA sequencing data demonstrated that macrophages are among the most abundant innate immune cells in peripheral blood mononuclear cells (PBMCs) and glandular tissues in pSS patients [4]. Additionally, research conducted using murine models of pSS suggests that macrophages may act as a mediator between CD4 + T cells and local tissue damage [5, 6].

In response to various stimuli, macrophages can be categorized into two distinct subtypes: classically activated M1 macrophages and alternatively activated M2 macrophages. The polarization of macrophages is regulated by a range of factors, including interferon (IFN), lipopolysaccharide (LPS), interleukin (IL), and noncoding RNAs. M1 macrophages, which are induced by LPS or IFN-γ, primarily secrete inflammatory cytokines such as tumor necrosis factor (TNF)-α, IL-6, IL-1β, and IL-23, thereby exerting pro-inflammatory effects. In contrast, M2 macrophages, which are activated by IL-4 or IL-13, play a role in resolving inflammation and promoting tissue regeneration by producing anti-inflammatory mediators such as IL-4, IL-10, and transforming growth factor (TGF)-β. Consequently, the identification of cytokines associated with specific phenotypes in peripheral blood can offer valuable insights into the state of macrophage polarization.

Macrophage polarization is implicated in the onset and progression of various inflammatory and autoimmune diseases, including systemic lupus erythematosus, rheumatoid arthritis and Sjogren’s syndrome [7–11]. Previous research has demonstrated that pSS patients exhibit significantly elevated levels of serum TNF-α, IL-6, and IL-12, which are closely linked to disease activity [12–14]. A recent study utilizing a rabbit model of pSS revealed an upregulation of pro-inflammatory M1-related markers and a downregulation of M2-related markers in lacrimal glands, suggesting the involvement of M1/M2 macrophage balance in the development of autoimmune dacryoadenitis [15]. However, the precise contribution of M1 and M2 macrophages in the context of pSS remains incompletely elucidated.

The objective of this investigation was to explore the role of macrophage polarization in the pathogenesis of pSS. In this study, we investigated the expression of M1/M2 macrophages in the peripheral blood of patients with pSS and healthy controls (HC) using enzyme-linked immuno sorbent assay (ELISA) and flow cytometry. Then, we examined the infiltration of M1/M2 macrophages in minor salivary gland samples through immunofluorescence. Furthermore, we conducted an analysis to determine the relationship between M1/M2 markers and some disease activity indicators in pSS.

## Materials and methods

### Patients and controls

The study was conducted at the department of rheumatology in the Second Affiliated Hospital Zhejiang University School of Medicine and was approved by the local ethics committee. Informed consent was obtained from all the participants prior to the sample collection.

Thirty female patients diagnosed with pSS were recruited. The enrolled patients met the 2016 American College of Rheumatology–European League against Rheumatism classification criteria for pSS [16]. Patients combined with other connective tissue disease, cancer, lymphoma, infection or other severe diseases were excluded. Peripheral venous blood samples (6 mL) were extracted from each patient for PBMCs and serum isolation. Minor salivary gland samples were abtained from 10 of these patients for immunofluorescence test. None of the patients had received any glucocorticoid or immunosuppressive agent treatment prior to specimen collection. The medical records of the patients were collected, including the severity of sicca symptoms, various clinical and laboratory parameters and systemic involvement of the patients. The disease activities of pSS patients were evaluated using Eular Sjogren’s syndrome disease activity index (ESSDAI).

Simultaneously, 30 female healthy controls matched for age were included in the study and peripheral venous blood samples were collected. In addition, minor salivary gland samples of non-pSS controls were from 10 subjects who presented with sicca symptoms and underwent minor salivary gland biopsy but didn’t fulfill the classification criteria for pSS.

### ELISA

Peripheral venous blood samples (2 ml) from each subject were collected in serum separation tubes and underwent centrifugation at 3000 rpm for 10 min to isolate serum. Serum samples were stored in -80℃ refrigerator. The serum concentration of M1 macrophage related cytokines (TNF-a, IL-6 and IL-23) and M2 macrophage related cytokines (IL-4, IL-10 and TGF-β) were measured by human ELISA kit (mlbio, Shanghai) in accordance with the instructions of the manufacturer.

### Flow cytometry

Peripheral venous blood samples (4 ml) were collected in EDTA-K2 anticoagulation tubes, and PBMCs were immediately isolated by Ficoll-Hypaque density gradient separation.

Cells were washed 2 times with cold PBS, scraped and collected by centrifugation and resuspended in FACS buffer (PBS supplemented with 0.5% bovine serum albumin and 5mM of EDTA). After Fc blocking, cells were stained with anti-CD86 (cat. no. 374,206, BioLegend, 1:200) and anti-CD206 (cat. no. 321,110, BioLegend, 1:200). Cells were analyzed on a BD LSRFortessa X-20 Cell Analyzer (BD Bioscience) with post-processing in FlowJo software (Tree star Inc). Cell populations were gated on forward and side scatter to select intact single cells. The gating strategy and a representative flow diagram is shown in Supplementary Figure S1.

### Immunofluorescence staining and microscopy

Minor salivary gland lobules were carefully harvested and placed into formalin fixative. Standard paraffin preparations were prepared and these were sectioned at 5-µm thickness. The paraffin sections of minor salivary gland tissues underwent routine dewaxing to water before being placed in a high-pressure 3% citric acid repair solution to repair antigens. Next, 3% hydrogen peroxide was added for a 10-min period. Afterwards, primary antibodies (anti-CD86 antibody and anti-CD206 antibody) were dropped onto the sections at a 1:100 dilution and left overnight at 4℃. The slides underwent three rounds of washing with PBS buffer, each lasting 5 min. Subsequently, a fluorescent secondary antibody was applied in drops and incubated at 37℃ for 30 min. The slides were then washed again with PBS buffer for 5 min, repeated three times before drops of 4,6-diamidino-2-phenylindole (DAPI) were applied, and incubated at room temperature for 10 min. The slides were rinsed with tap water and then sealed with a water-soluble sealant. Finally, samples were visualized and photographed with Leica STELLARIS 5 (Germany). Using imaging software (Image J), an analysis of densitometry and fluorescence intensity was performed. Results were presented as mean optical density values which were equal to an integrated optical density per unit area (mm^2^).

### Statistical analysis

Statistical analyses were performed using Graphpad Prism 10.0 software. Categorical variables were presented as numbers or percentages. Continuous variables were presented as mean ± standard deviation (SD) or median (interquartile range [IQR]). Data were tested for normality using the D’Agostino & Pearson test. Unpaired 2-tailed Student’s t-test was used to compare quantitative data consistent with a normal distribution, and Mann–Whitney U test was used to compare data not consistent with a normal distribution. Two-sided *p*-value < 0.05 was considered as statistically significant.

## Results

### Clinical characteristics of subjects

The characteristics of the patients and controls are summarized in Table 1. All the patients, health controls and non-pSS controls were female, with mean age 43.4 ± 12.2, 41.1 ± 9.9 and 46.6 ± 12.8 years respectively. Out of the 30 enrolled pSS patients, 25 tested positive for anti-SSA/Ro antibodies, and 6 had anti-SSB/La antibodies. Histological findings of minor salivary glands were graded on the Chisholm–Mason scale, with grade 3(1 focus of lymphocytes per 4 mm^2^ of minor salivary tissue) and grade 4 (more than 1 focus of lymphocytes per 4 mm^2^ of minor salivary tissue) being regarded as diagnostic for pSS [17]. Meanwhile, grade 1 (mild lymphocytic infiltration) and grade 2 (moderate lymphocytic infiltration) were considered not meet the criteria for pSS. A total of 20 pSS patients underwent minor salivary gland biopsy, and all of those met the criteria for pSS. In the pSS group, the median ESSDAI score was 3, the median percentage of B cells in lymphocytes was 17.0%, and the median serum immune globulin G (IgG) level was 18.5 g/L. The health control group did not have any available immunological laboratory data. The patients in the non-pSS control group tested negative for antinuclear antibodies, and their median serum IgG level was 10.8 g/L. All of the patients in non-pSS control group didn’t meet the criteria for pSS.

**Table 1:** The demographic, clinical, and laboratory characteristics of the patients and controls

	pSS (*n* = 30)	HC (*n* = 30)	non-pSS (*n* = 10)
Female, N (%)	30 (100)	30 (100)	10 (100)
Age (years), mean ± SD	43.4 ± 12.2	41.1 ± 9.9	46.6 ± 12.8
Duration of sicca symptoms, median (IQR) years	3.0 (1.5-8.0)	/	2.5 (1.0-6.5)
Oral dryness (VAS,1–10), median (IQR)	5.5 (2.5-6.0)	/	2.0 (0-4.5)
Ocular dryness (VAS,1–10), median (IQR)	3.0 (1.0-5.5)	/	1.5 (0-3.5)
Labial salivary gland biopsies, N	20	/	10
Grade1, N	0	/	7
Grade2, N	0	/	3
Grade3, N	9	/	0
Grade4, N	11	/	0
IgG (g/L), median (IQR)	18.5 (15.3–23.8)	/	10.8 (9.3–11.8)
C3 (g/L), mean ± SD	0.95 ± 0.23	/	1.01 ± 0.29
C4 (g/L), mean ± SD	209.5 ± 92.7	/	205.1 ± 83.5
ESR (mm/h), median (IQR)	27 (13–48)	/	12 (5–18)
CRP (mg/L), median (IQR)	3.9 (3.1–7.5)	/	3.6 (2.8–7.3)
RF positivity, N (%)	14 (46.7)	/	1 (10.0)
Anti-SSA positivity, N (%)	25 (83.3)	/	0 (0)
Anti-SSB positivity, N(%)	6 (20.0)	/	0 (0)
B cell, %. mean ± SD	17.0 ± 7.2	/	/
ESSDAI score, median (IQR)	3 (1–4)	/	/
Organ involvement			
Interstitial lung disease, N (%)	5 (16.7)	/	/
Haematologic, N (%)	6 (20.0)	/	/
Renal, N (%)	2 (6.7)	/	/
Parotitis, N (%)	5 (16.7)	/	/
Arthritis, N (%)	4 (13.3)	/	/
Peripheral neuropathy, N (%)	1 (3.3)	/	/

pSS: primary Sjogren’s syndrome; HC: health control; SD: standard deviation; IQR: inter-quartile range; VAS: visual analogue scale; IgG: immunoglobulin G; C3/C4: complement 3/4; ESR: erythrocyte sedimentation rate; CRP: C- reactive protein; RF: rheumatoid factor; ESSDAI: Eular Sjogren’s syndrome disease activity index

### Serum levels of cytokines in pSS patients and health controls

**Fig. 1:**
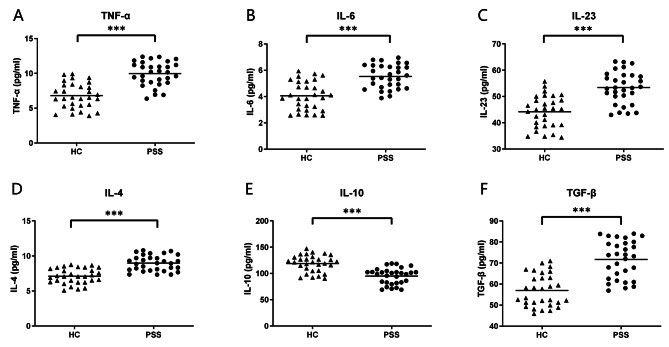
Serum levels of cytokines in pSS patients and health controls. ***: *p* < 0.001

**Fig. 2:**
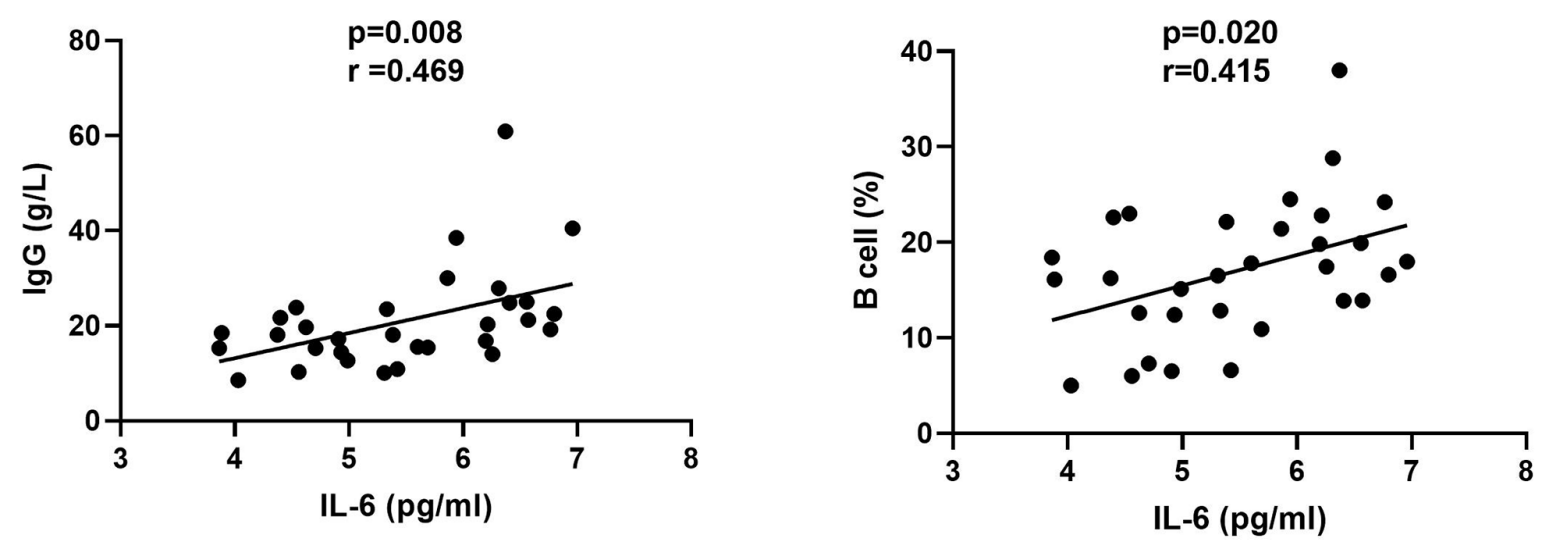
The correlation between serum levels of IL-6 and that of IgG as well as percentages of CD19 + B cells in lymphocytes of pSS

The concentrations of various serum cytokines were measured in 30 pSS patients and 30 healthy controls using ELISA. Figure 1 demonstrates that the levels of TNF- α, IL-6, IL-23, IL-4 and TGF-β were significantly higher in the pSS group compared to the healthy controls, while the levels of IL-10 were lower. Subsequently, an examination was conducted to assess the relationship between these cytokines and various disease activity indicators in patients with pSS, including IgG, erythrocyte sedimentation rate (ESR), rheumatoid factor (RF), number of CD19^+^ B cells and ESSDAI score. The results revealed a significant positive correlation between serum levels of IL-6 and that of IgG (*p* = 0.008, *r* = 0.469) as well as percentages of CD19 + B cells in lymphocytes (*p* = 0.020, *r* = 0.415) (Fig. 2). While no association was observed between the other cytokines and these indicators.

### Numbers of M1 and M2 macrophages in the peripheral blood of pSS patients and healthy controls

**Fig. 3:**
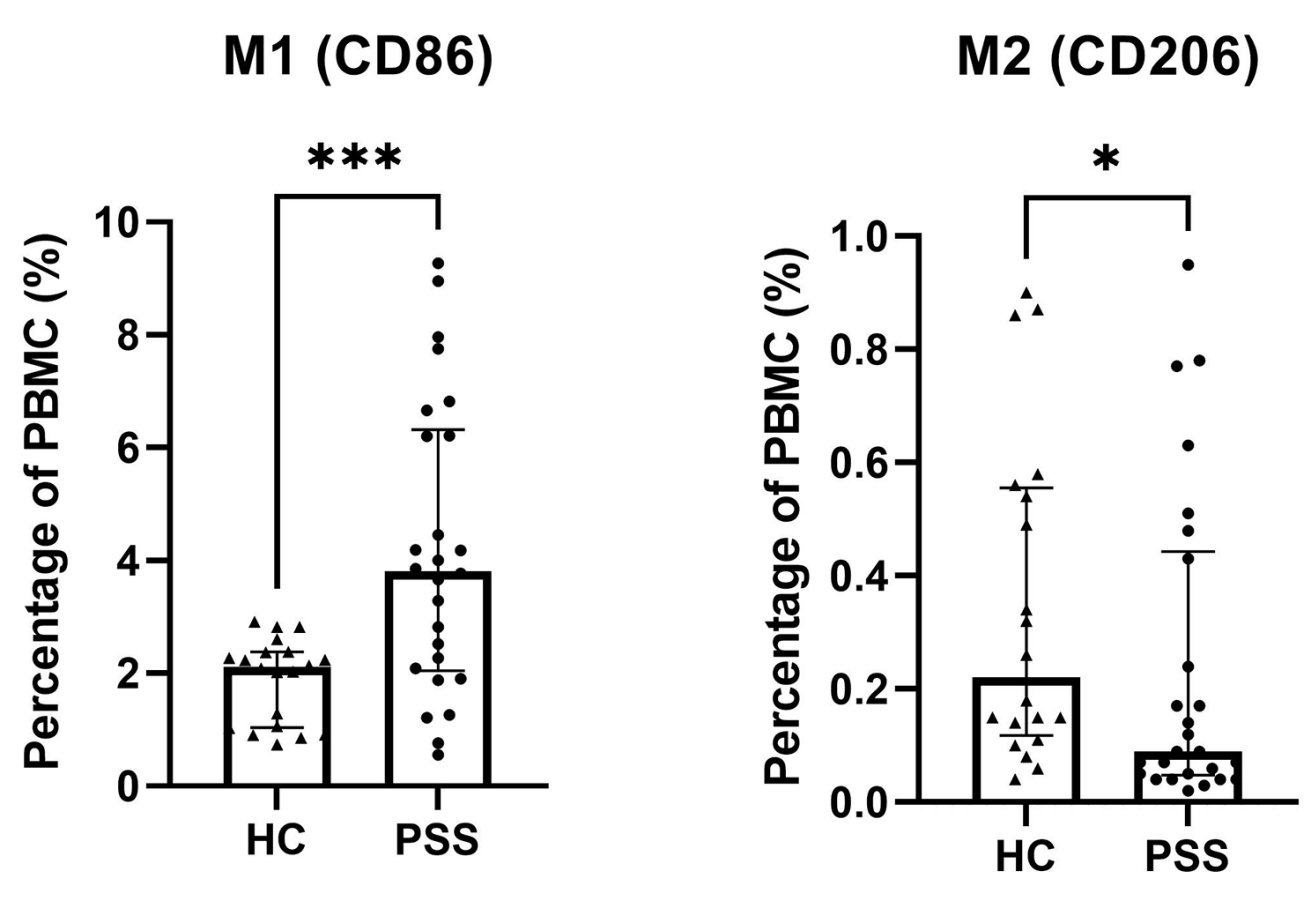
Numbers of M1 and M2 macrophages in the peripheral blood of pSS patients and healthy controls. *: *p* < 0.05; ***: *p* < 0.001

**Fig. 4:**
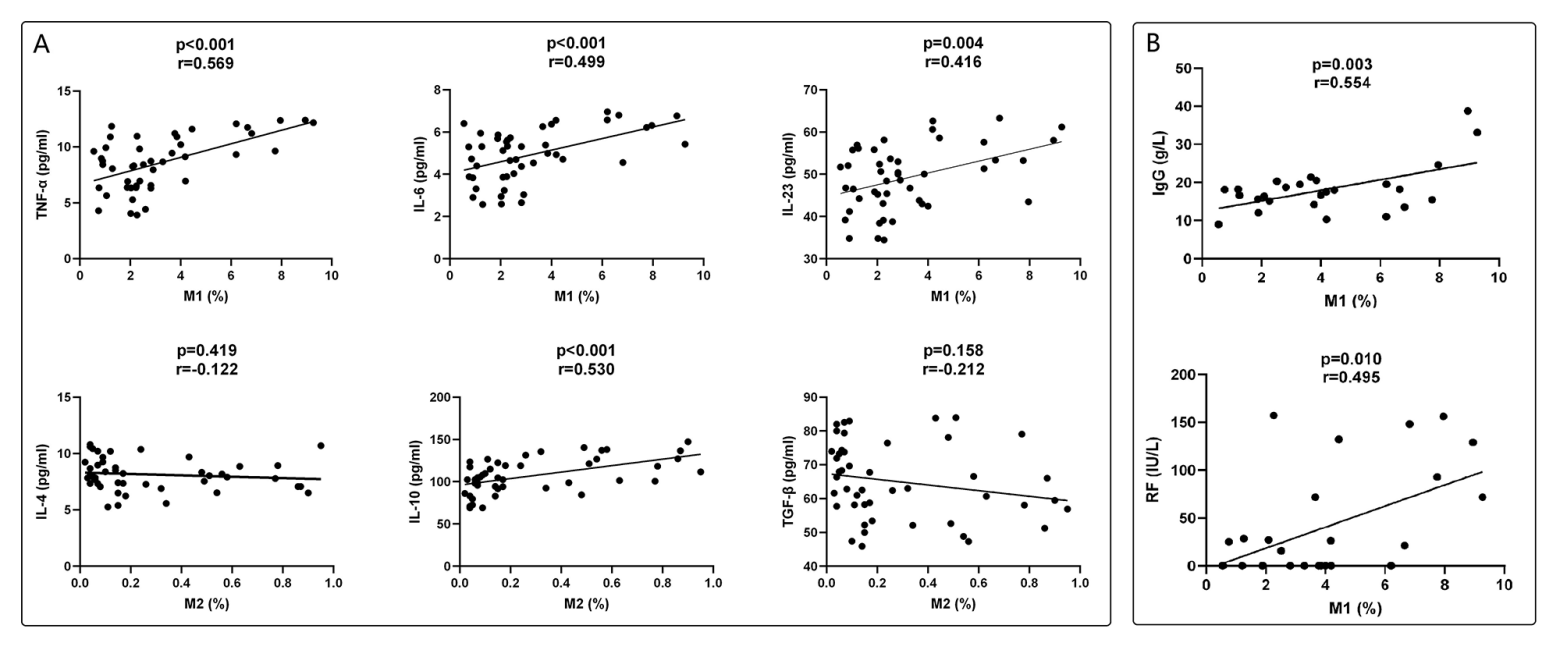
(**A**) The correlation between proportions of M1/M2 macrophages in PBMCs and serum levels of cytokines; (**B**) The correlation between proportions of M1 macrophages in PBMCs and serum levels of IgG and RF

Flow cytometry was conducted to analyze the quantities of peripheral CD86 + M1 and CD206 + M2 macrophages in a cohort of 26 pSS patients and 20 healthy controls (Fig. 3). Notably, the median percentage of M1 macrophages in PBMCs was found to be 3.81(2.04–6.32)% in the pSS group, which was significantly higher compared to 2.12(1.04–2.38)% in the control group (*p* < 0.001). The median percentage of M2 macrophages in PBMCs in the pSS group was lower than that in the control group (0.09[0.05–0.44]% vs. 0.22[0.12–0.56]%, *p* = 0.039). Next, we investigated the relationship between the numbers of M1/M2 macrophages and the serum levels of cytokines (Fig. 4A) as well as various disease activity indicators (IgG, ESR, RF, number of CD19^+^ B cells and ESSDAI score) (Fig. 4B). Our findings revealed a significant positive correlation between the proportions of M1 macrophages in PBMCs and the serum levels of TNF-α (*p <* 0.001, *r* = 0.569), IL-6 (*p <* 0.001, *r* = 0.499), IL-23 (*p* = 0.004, *r* = 0.416), IgG (*p* = 0.003, *r* = 0.554) and RF (*p* = 0.010, *r* = 0.495). No significant correlation was observed between the numbers of M1 macrophages and the levels of ESR, CD19^+^ B cells and ESSDAI scores. However, the numbers of M2 macrophages exhibited a positive correlation only with IL-10 levels (*p <* 0.001, *r* = 0.453), but no correlation with IL-4 and TGF-β. Additionally, no relationships were found between numbers of M2 macrophages and these disease activity indicators.

**Expression of M1 and M2 macrophages in the minor salivary glands of pSS patients and non-pSS controls**.

**Fig. 5:**
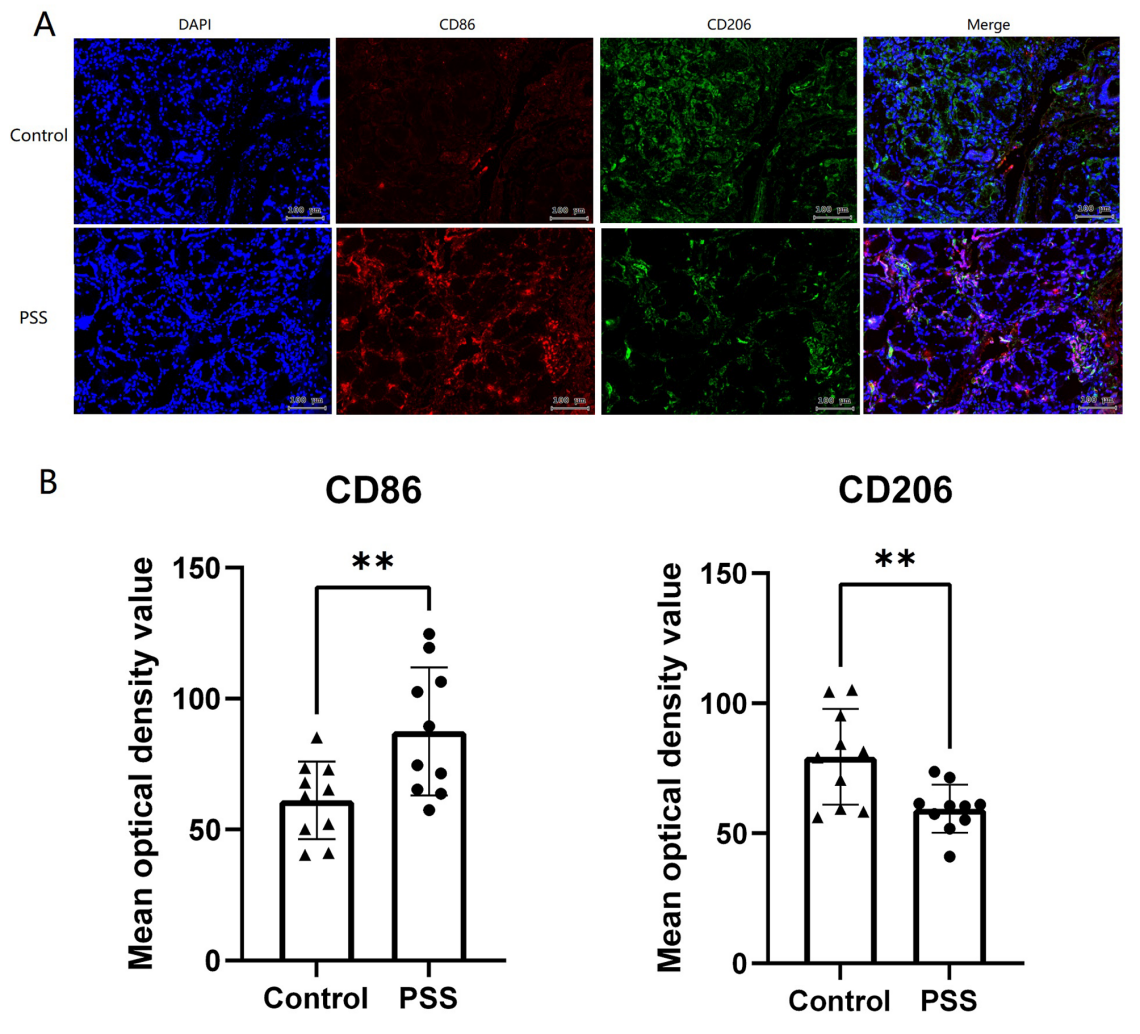
Expression of CD86 + M1 and CD206 + M2 macrophages in the minor salivary glands of pSS patients and non-pSS controls. A: Immunofluorescence photographs of CD86 and CD206 in the minor salivary glands; B: Statistical plots of mean optical density values of CD86 and CD206 in the minor salivary glands; DAPI fluorescence is shown in blue, CD86 immunofluorescence is shown in red and CD206 immunofluorescence is shown in green

Using an immunofluorescence technique, the expression of CD86 + M1 and CD206 + M2 macrophages in minor salivary gland tissues from 10 patients with pSS and 10 non-pSS controls was evaluated. Figure 5 illustrates that the expression of CD86 + M1 macrophages was significantly higher in pSS patients compared to non-pSS controls, while the expression of CD206 + M2 macrophages was significantly lower. These findings provide support for the predominance of M1 macrophages in the minor salivary glands of pSS patients.

## Discussion

The role of macrophage polarization in the pathogenesis of various autoimmune inflammatory diseases, particularly systemic lupus erythematosus [8] and rheumatoid arthritis [9], has been well-established. This study demonstrates the presence of an imbalanced expression of M1/M2 macrophages in patients with pSS.

Previous research has demonstrated a significant increase in the expression of pro-inflammatory cytokines, such as IL-6, IL-17, IL-23, TNF-α and IL-1β, both in serum and minor salivary glands of patients with pSS [18]. And it was reported that serum levels of TNF-α and IL-6 were associated with multiple activity indicators of pSS [14]. These cytokines are primarily secreted by M1 macrophages, suggesting a predominant polarization of M1 macrophages in pSS patients. Moreover, a study has provided clarification that peripheral blood monocytes derived from patients with pSS exhibit a higher secretion of IL-6 when stimulated by IFN-γ, a known inducer of M1 polarization [19]. This study also demonstrated a significant increase in the numbers of M1 macrophages in peripheral blood and elevated levels of M1-related pro-inflammatory cytokines (IL-6, IL-23 and TNF-α) in pSS patients, compared to healthy individuals. These findings confirm the activation of M1 macrophages in pSS patients. Further analysis revealed a significant positive correlation between the levels of M1 macrophages in peripheral blood and the serum levels of IgG and RF. Additionally, there was a notable positive correlation observed between serum levels of IL-6 and that of IgG as well as the percentage of CD19 + B cells in lymphocytes. As is known, the presence of elevated CD19 + B lymphocytes and hypergammaglobulinaemia are indicative of immune system hyperactivation in patients with pSS and are closely linked to disease activity. RF also serves as an indicator of disease activity in pSS. These correlations imply that the M1 polarization of macrophages may have a significant role in the development of pSS and may be associated with disease activity.

In a previous study, the presence of M2 macrophages in the salivary gland of patients with pSS was found to be inversely correlated with the severity of inflammatory lesions [20]. This suggests that M2 macrophages may play a role in the anti-inflammatory functions within pSS lesions. Furthermore, higher levels of IL-10 and IL-4 were observed in pSS patients, particularly in those with elevated IgG levels [12]. IL-10, which is secreted by macrophages and regulatory T cells, regulates the synthesis of pro-inflammatory cytokines by macrophages and inhibits the production of Th1 cytokines. IL-4 suppresses the proliferation of Th1 cells and inhibits the synthesis of Th1 cytokines. TGF-β, known for its ability to promote M2 macrophage polarization and induce fibrosis [21], exhibits a protective and anti-inflammatory effect in autoimmune processes. The expression patterns of TGF-β in the peripheral blood and salivary gland of patients with pSS are variable and complex [22, 23]. This study observed a downregulation of M2 macrophages in the peripheral blood of pSS patients, although significant individual differences were noted. Serum levels of IL-10 were downregulated in accordance with the changes in M2 macrophages. Conversely, serum levels of IL-4 and TGF-β were upregulated and showed no correlation with the numbers of M2 macrophages.

Given that pSS is a chronic and progressive disease characterized by varying cytokine expression profiles depending on different disease courses. The incongruous findings mentioned above may be attributed to the disparate disease courses among subjects in different studies. The process of macrophage polarization is dynamic and reversible. M1 macrophages polarization is predominant during the initial stages of pSS. These M1 macrophages produce inflammatory factors, thereby assuming a proinflammatory role and subsequently activating CD4 + T cells to differentiate into Th1 cells, ultimately leading to inflammation of the exocrine glands [24]. It is evident that M2 macrophages contributed to the regression of inflammation and tissue regeneration mainly in the late stage of pSS, when chronic inflammation progresses to irreversible salivary gland fibrosis. However, our study has some limitations due to the absence of sampling at different stages of the disease. These findings suggest that the M2 polarization of macrophages undergoes more intricate changes in pSS.

It is well-established that infiltrating cells in minor salivary gland lesions of pSS patients primarily consist of T and B lymphocytes. Additionally, studies have demonstrated the presence of other infiltrating cells such as macrophages, dendritic cells, and natural killer cells in minor salivary gland [25, 26]. The available evidence indicates that the composition of infiltrates varies depending on the severity of the lesion. The presence of B cells and macrophages showed a positive correlation with the degree of infiltration and the biopsy focus score. Additionally, a higher infiltration of macrophages was associated with cryoglobulinemia, parenchymal-organ involvement, and lymphoma [2]. This study discovered that the predominant type of macrophages infiltrating the minor salivary glands of pSS patients were M1 macrophages. These findings suggest that M1 macrophages, in interaction with T and B lymphocytes, may have a significant role in the damage to the glands in pSS patients. In contrast, a reduction in M2 macrophages was observed in the minor salivary glands of patients with pSS, potentially contributing to the chronic inflammatory progression of the glands, as previously mentioned [20].

The function of macrophages is regulated by interferon regulatory transcription factor (IRF)/signal transducer and activator of transcription (STAT) signaling pathways. Activation of nuclear factor (NF)-κB and STAT1 by IFN and Toll-like receptor (TLR) signaling predominantly promotes polarization of macrophages towards the M1 phenotype, characterized by cytotoxic and inflammatory functions. Conversely, activation of STAT3 and STAT6 by IL-4 and IL-13 predominantly leads to polarization of macrophages towards the M2 phenotype, associated with immune suppression and tumor progression [27]. The role of innate immune regulation, particularly mediated by the type I IFN signature, is widely recognized as crucial in the pathogenesis of pSS. A comprehensive RNA-sequencing analysis revealed differentially expressed genes in M1 macrophages of pSS patients, with the most significant enrichment observed in type I IFN signaling [28]. Furthermore, an vitro study confirmed that type I IFN signaling induces polarization of macrophages towards the M1 phenotype [29]. Consequently, it is plausible to suggest that the polarization of macrophages towards the M1 phenotype serves as a link between the activation of type I IFN signaling and the onset of pSS.

The immune responses encompass intricate cellular and molecular processes. Although it may be an oversimplification to categorize complex diseases like pSS solely based on M1 or M2 macrophage polarization, this conceptual framework facilitates a deeper comprehension of the underlying mechanisms driving the disease’s pathogenesis.

## Conclusion

This study reveals the presence of an significant imbalance in M1/M2 macrophages in pSS patients. The M1 polarization of macrophages may play an central role in the pathogenesis of pSS. Consequently, modulating this imbalance in macrophage polarization could potentially serve as an efficacious therapeutic approach to ameliorate the onset and progression of pSS. However, the precise molecular mechanism about the imbalance of M1/M2 macrophages in pSS remains to be further explored.

### Electronic supplementary material

Below is the link to the electronic supplementary material.

Supplementary Material 1

## Data Availability

No datasets were generated or analysed during the current study.

## Abbreviations

DAPI: 4,6-diamidino-2-phenylindole
ELISA: enzyme-linked immuno sorbent assay
ESR: erythrocyte sedimentation rate
ESSDAI: Eular Sjogren’s syndrome disease activity index
HC: healthy controls
IFN: Interferon
IgG: immune globulin G
IL: interleukin
IQR: interquartile range
IRF: interferon regulatory transcription factor
LPS: lipopolysaccharide
NF: nuclear factor
PBMCs: peripheral blood mononuclear cells
pSS: Primary Sjogren’s syndrome
RF: rheumatoid factor
SD: standard deviation
STAT: signal transducer and activator of transcription
TGF: transforming growth fact
TNF: tumor necrosis factor
