# Supplementary figures and images for: The role of M1/M2 macrophage polarization in primary Sjogren’s syndrome
Source: Arthritis Res Ther. 2024 May 14;26:101. doi: 10.1186/s13075-024-03340-7 (PMC11092035; doi:10.1186/s13075-024-03340-7)

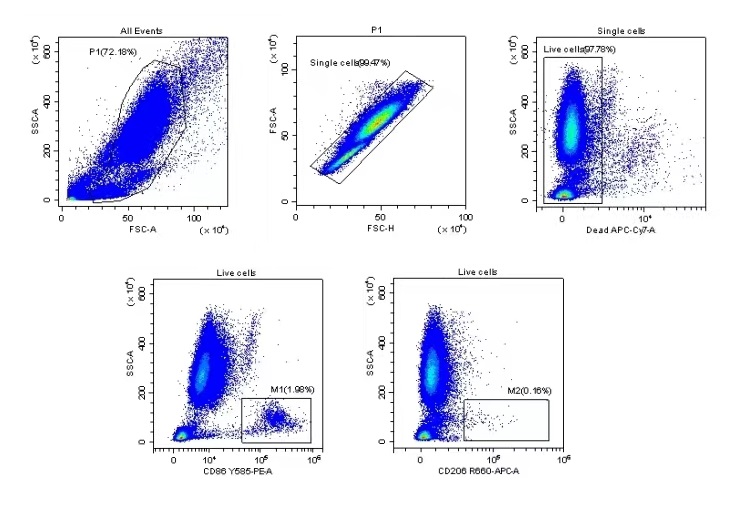

Supplement: Supplementary file 1 — Supplementary Material 1 [file 13075_2024_3340_MOESM1_ESM.jpg]
